# Application of a long short-term memory neural network: a burgeoning method of deep learning in forecasting HIV incidence in Guangxi, China

**DOI:** 10.1017/S095026881900075X

**Published:** 2019-05-09

**Authors:** G. Wang, W. Wei, J. Jiang, C. Ning, H. Chen, J. Huang, B. Liang, N. Zang, Y. Liao, R. Chen, J. Lai, O. Zhou, J. Han, H. Liang, L. Ye

**Affiliations:** 1 Guangxi Collaborative Innovation Center for Biomedicine, Life Science Institute, Guangxi Medical University, Nanning 530021, Guangxi, China; 2 Guangxi Key Laboratory of AIDS Prevention and Treatment & Guangxi Universities Key Laboratory of Prevention and Control of Highly Prevalent Disease, School of Public Health, Guangxi Medical University, Nanning 530021, Guangxi, China; 3 Geriatrics Digestion Department of Internal Medicine, The First Affiliated Hospital of Guangxi Medical University, Nanning 530021, Guangxi, China

**Keywords:** ARIMA model, HIV, incidence, LSTM model, prediction

## Abstract

Guangxi, a province in southwestern China, has the second highest reported number of HIV/AIDS cases in China. This study aimed to develop an accurate and effective model to describe the tendency of HIV and to predict its incidence in Guangxi. HIV incidence data of Guangxi from 2005 to 2016 were obtained from the database of the Chinese Center for Disease Control and Prevention. Long short-term memory (LSTM) neural network models, autoregressive integrated moving average (ARIMA) models, generalised regression neural network (GRNN) models and exponential smoothing (ES) were used to fit the incidence data. Data from 2015 and 2016 were used to validate the most suitable models. The model performances were evaluated by evaluating metrics, including mean square error (MSE), root mean square error, mean absolute error and mean absolute percentage error. The LSTM model had the lowest MSE when the N value (time step) was 12. The most appropriate ARIMA models for incidence in 2015 and 2016 were ARIMA (1, 1, 2) (0, 1, 2)_12_ and ARIMA (2, 1, 0) (1, 1, 2)_12_, respectively. The accuracy of GRNN and ES models in forecasting HIV incidence in Guangxi was relatively poor. Four performance metrics of the LSTM model were all lower than the ARIMA, GRNN and ES models. The LSTM model was more effective than other time-series models and is important for the monitoring and control of local HIV epidemics.

## Introduction

HIV/AIDS has brought tremendous challenges to global public health and life quality of humankind worldwide [1]. In China, a total of 769 175 patients living with HIV/AIDS were reported as of January 2018, of which 445 716 cases survived with HIV infection, 323 459 cases were AIDS patients and there were 241 519 deaths [2]. As an HIV-hit region, Guangxi Zhuang Autonomous Region, a province in southwestern China, has seen an increasing HIV incidence in recent years, and it has become a major public health problem in Guangxi [3, 4]. Therefore, corresponding intervention strategies as well as methods against local HIV/AIDS epidemics need urgently to be explored and developed. As the World Health Organization (WHO) has advocated, effective disease surveillance is dependent on effective disease control [5]. Disease surveillance can provide a decision-making basis for disease prevention and control, and can also evaluate the feasibility and effectiveness of prevention and control interventions. Nevertheless, disease surveillance data have a weakness, namely hysteresis, which means that disease surveillance reflects just the current status but has little capacity to forecast future epidemics [6]. However, the government needs an accurate prediction in HIV incidence to formulate targeted interventions. Therefore, establishing high-precision prediction models is important for the monitoring and control of local HIV epidemics.

Disease forecasting plays an important role in disease prevention. Many mathematical models including linear regression, generalised linear regression, non-linear regression, decision trees, neural networks, etc. [7–9], have been developed to predict the incidence of infectious diseases. The autoregressive integrated moving average (ARIMA) model is a classical model based on linear theory. The ARIMA model was first proposed by Box and Jenkins in the early 1970s, to predict future tendency using the past and present data of time series [10]. ARIMA transforms the non-stationary time series into a stationary time series, and then establishes a regression model with the lag value of dependent variable, the present value and lag values of the random error [11, 12]. However, the ARIMA model mainly captures a linear relationship, which may produce a large deviation for non-linear or unstable information. The generalised regression neural network (GRNN) model, which has a strong non-linear mapping ability and learning speed, has been widely used in disease prediction, modelling and estimating [13, 14]. The effect of prediction is excellent when the data series is small and unstable. Furthermore, exponential smoothing (ES), a widespread method, is also a general model for analysing time series such as production forecasts and short-to-medium-term economic development trends [15].

In recent years, the rapid development of deep learning methods has provided an alternative approach for disease prediction [16]. The long short-term memory (LSTM) model, proposed first by Sepp Hochreiter and Jurgen Schmidhuber in 1997, is a neural network based on new deep learning of a recurrent neural network (RNN) [17]. Using multi-layer and complex neural networks close to the real values, a backward propagation algorithm is used to continually shrink the fitting error [18]. The complex mathematical modelling and solving processes can be skipped by a cyclic neural network model based on LSTM, which is an extension of an RNN and suitable to solve the problem of correlation in time series. The LSTM includes three types of gates: the forget gate, the input gate and the output gate. These gates can be opened or closed to judge whether the result reaches the threshold and thus is to be included in the current calculation of this layer. Because of the gates, the useful data in the time series are kept, and the useless data discarded, so that the interference of useless data can be avoided and the model is more accurate in processing time series. Also, the LSTM model has been widely used in many fields, such as image recognition, speech recognition, etc. [19, 20]. Nevertheless, so far LSTM has rarely been used in forecasting infectious diseases, especially HIV infection. In this study, we used LSTM, ARIMA, GRNN and ES models to estimate HIV incidence in Guangxi, China, and to explore which model is the best and most precise for local HIV incidence prediction.

## Methods

### Ethical approval

The data came from a public-access secondary database. Formal ethical approval was not required for this study.

### Data source

Data of HIV incidence in Guangxi from January 2005 to December 2016 were obtained from the public-access database of the Chinese Center for Disease Control and Prevention (CDC, http://www.phsciencedata.cn). All HIV cases of Guangxi were confirmed by clinical and laboratory tests, which were reported to the Chinese CDC within 12 h of confirmation via an Internet-based national disease-reporting system.

### Construction of the ARIMA model

The ARIMA model is the most classical and commonly used model in non-stationary time-series analysis. We explored the optimal parameter values for the model using Eview 8.0 software. Construction of the ARIMA model is based on the formula as follows [11]: Φ(B)(1 − B)^*d*^*Xτ* = *θ*(B)*ε_τ_*

In this formula, *Xt* represents the non-stationary values of a sequence at time*t*; *εt* is the white noise; *d* means differential times; *B* represents the backward shift operator; and *θ*(*B*) represents the moving-average operator. The ARIMA model based on the season is described as ARIMA (*p*, *d*, *q*)(*P*, *D*, *Q*)*s*, where *p* is the number of autoregressive order; *d* is the degree of difference; *q* is the sliding average order; *P* is the seasonal autoregressive order number; *D* is the degree of seasonal difference; *Q* is the seasonal sliding average order; and *s* is the number of cycles [11].

Establishing an ARIMA model consists of four steps, as follows [21]:

Initially, time-series data were obtained from the data system.

Second, data were drawn to observe whether the time series was stationary. With regard to non-stationary time series, the *d* order difference operation was first performed, which was converted into a stationary time series.

Thirdly, the autocorrelation function (ACF) analysis and the partial autocorrelation function (PACF) analyses were employed to determine the possible values of *p*, *q*, *P* and *q*. Then, the models in which the parametric tests were not statistically significant (*P* > 0.05) were excluded, and the residual tests showed the non-white noise sequence using the Box–Jenkins *Q* test.

Finally, the Akaike information criterion (AIC) and Schwarz Bayesian information criterion (SBC) were used to determine the optimal model. The model with the lowest AIC and SBC values was considered as the optimal model. If the AIC and SBC values were nearly equal, the model with the higher *R*^2^ value was selected as the optimal one.

### Construction of the LSTM model

**Fig. 1.**
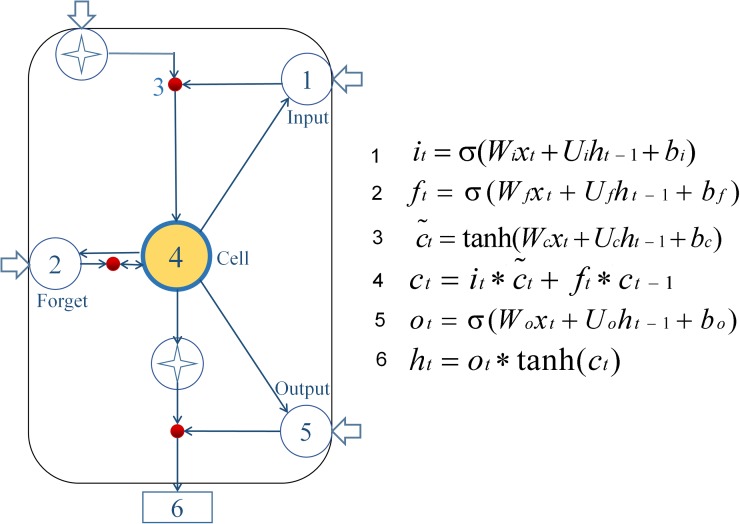
Diagram of LSTM neural network pattern. Input gate (*i_t_*) determines which information needs to be updated in the unit state; the forgetting gate (*f_t_*) controls information which needs to be discarded from the unit state; then input gate and a vector 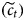 are created by Tanh to determine which new information is stored in the unit state to update the old unit state, and turn into the new unit state (*c_t_*). Finally, cell state information is filtered with the output gate (*o_t_*) to update the hidden state (*h_t_*), which is the output of the LSTM cell.

The LSTM model is virtually a neural network based on new deep learning by the RNN [17]. In this study, we used Anaconda based on Python 3.5 programming to develop the LSTM model. The calculation node [22] is shown in Figure 1.

In the formulas, the input gate (*i*_*t*_) determines which information needs to be updated in the unit state; and the forget gate (*ft*) controls information which needs to be discarded from the unit state. Next, the input gate and a vector 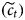 are created by Tanh to determine which new information is stored in the unit state to update the old unit state, and turn it into the new unit state (*c_t_*). Finally, cell state information is filtered with the output gate (*o_t_*) to update the hidden state (*h_t_*), which is the output of the LSTM cell [23, 24]. Briefly, the LSTM designed a structure to remove or add elements to the cell state called gates. The information was selected by these gates, which are composed of a sigmoid neural network layer and a scalar multiplication operation. The sigmoid layer outputs a value between 0 and 1, indicating how many components could pass, 0 means ‘no information can pass’, while 1 means ‘all information can pass’. Each gate is responsible for different tasks, and the forget gate is responsible for deciding how many cell states are reserved from the previous moment to the current moment. The input gate is responsible for deciding how many cell states are reserved from the current moment to the current moment; and the output gate is responsible for determining how much output the cell state has at the current time. In these formulas, the *W* matrices show the weight applied to the current input, the *U* matrices represent the weight applied to the previous hidden state, the *b* vectors are biases for each layer and *σ* is the sigmoid function. Establishing the LSTM model consisted of three steps:

Initially, the raw data were divided into two parts: data of the last 2 years were considered as the test set, and the rest data were used as a training set. The training samples were used to build the model and to discover potential relationships in the data, and the test samples were used to evaluate the predictive power of the model constructed by the training set.

Subsequently, a series of LSTM models were constructed using *N* values which were the time steps. For example, if the time step was 20, the value of the 21st data was predicted with the last 20 data as input. The model with the lowest mean square error (MSE) was considered the optimal model.

Finally, the incidence was predicted by the optimal model for the assignment of the lowest MSE.

### Construction of the GRNN model

The GRNN model, first proposed and developed by Specht [25], has a strong non-linear mapping ability and learning speed. The predictive effect is excellent when the data series is small and unstable data are also solved. The GRNN consists of four layers: an input layer, a pattern layer, a summation layer and an output layer [26, 27]. The input layer inputs sample data and the pattern layer calculates the Gauss value for each of the test and training samples. The summation layer consists of two nodes, the first is the output sum of each hidden layer node, and the second is the weighted sum of proleptic results and each hidden layer node. The output layer outputs the result of dividing two nodes from the third layer [28]. The method and steps have been previously described in detail [29].

### Construction of the ES model

The ES model is also a general method for analysing time series [15, 30]. It has a widespread use in the production of forecasting and also used for short-to-medium-term economic development trends. As a special form of the weighted average method, the ES method does not need to conduct a quantitative research on the internal factors and relations of systems, but to observe valuable information from the data itself. Establishing an ES model consists of three steps as follows [31]:

Initially, determining the initial values. If the time series had more original data, the original data were used to replace the initial value of the ES method; if the time series had fewer data, the average value of the original data was taken as the initial value.

Subsequently, selecting the smoothing factor *α* and calculating the ES values. The value of *α* determines the smoothing level and response speed to the difference between the predicted and actual values. When the time series was relatively stable, a smaller smoothing factor was obtained; otherwise, a larger smoothing factor was adopted.

Finally, the smoothing factor, which a minimised MSE, was determined and the predicted values were obtained.

### Patient and public involvement

Neither was involved.

## Results

### Construction of ARIMA models

**Fig. 2.**
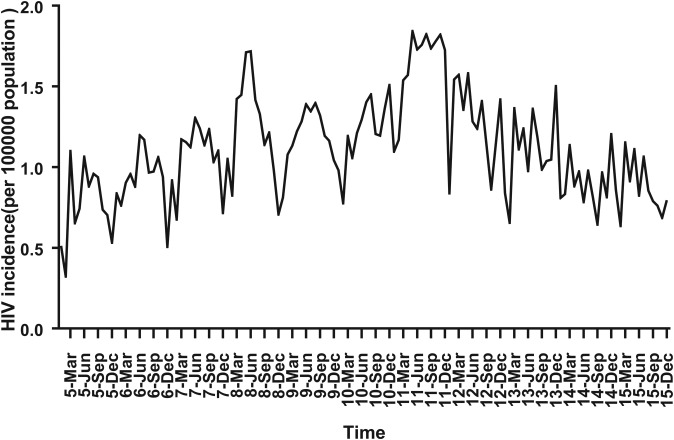
Monthly incidence of HIV in Guangxi, China (from January 2005 to December 2015). According to the trend section, it can be found that the incidence of HIV shows seasonal tendency (*s* = 12). From 2005 to 2011, the HIV incidence in Guangxi was increasing slowly, and the epidemic situation in 2011–2016 showed a seasonal slow decline.

For ARIMA model construction, monthly HIV incidence from January 2005 to December 2014 was used; for testing the predictive ability of these models, HIV incidence in 2015 or 2016 was used. Figure 2 shows the monthly HIV incidence from January 2005 to December 2015 in Guangxi, China. Overall, the HIV incidence exhibited a seasonal tendency (*s* = 12) (Fig. 2). From 2005 to 2011, the HIV incidence in Guangxi increased slowly, but the epidemic situation from 2011 to 2015 showed a slow and seasonal decline, which meant that the time series was not stationary. Thus, the data were processed by a log transformation with non-seasonal (*d* = 1) and seasonal difference (*D* = 1), to remove numerical instabilities. After data processing, the ADF test showed a statistically significant result (*P* < 0.0001), indicating that the time sequence was stationary (Supplementary Table S1).

The ACF and PACF analyses were then used to determine the parameters of the ARIMA models (Supplementary Fig. S1). According to the results of the ACF and PACF analyses (however, some models were not tested by parameters or residuals), three appropriate models, namely, ARIMA (2, 1, 0) (1, 1, 2)_12_, ARIMA (2, 0, 1) (0, 1, 2)_12_ and ARIMA (0, 1, 0) (2, 1, 2)_12_, were identified to predict the HIV incidence in 2016. These three models had approximate AIC and SBC values (Supplementary Table S2). However, the model ARIMA (2, 1, 0) (1, 1, 2)_12_ had a higher *R*^2^ than the other two, and it was selected as the optimal model. Supplementary Tables S3 and S4 list the parameter test results of the best prediction model, which showed the white noise sequence in 2015 and 2016, respectively. The forecasting curve of the optimal ARIMA model and the actual HIV incidence curve are shown in Figure 4.

### Construction of LSTM models

**Fig. 3.**
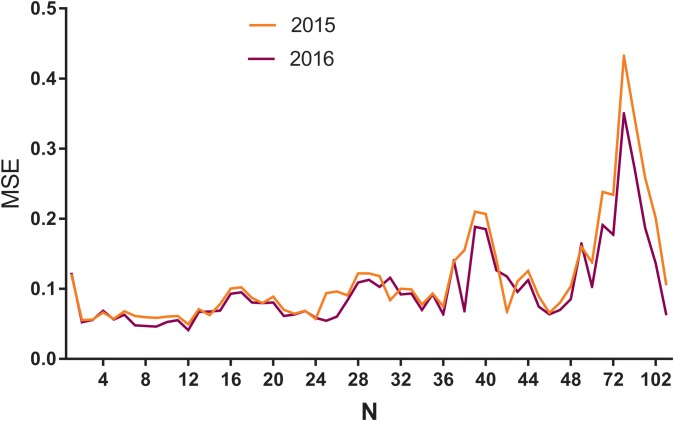
The MSE of LSTM models with different ***N*** values using HIV incidence in 2015 and 2016. MSE, mean square error; *N*: the number of input to the LSTM model. The yellow line means *N* value and corresponding MSE in 2015, while the purple line means *N* and MSE in 2016. As can be seen from the figure, when the *N* was 12, the model had the minimum MSE in 2015 and 2016.

**Fig. 4.**
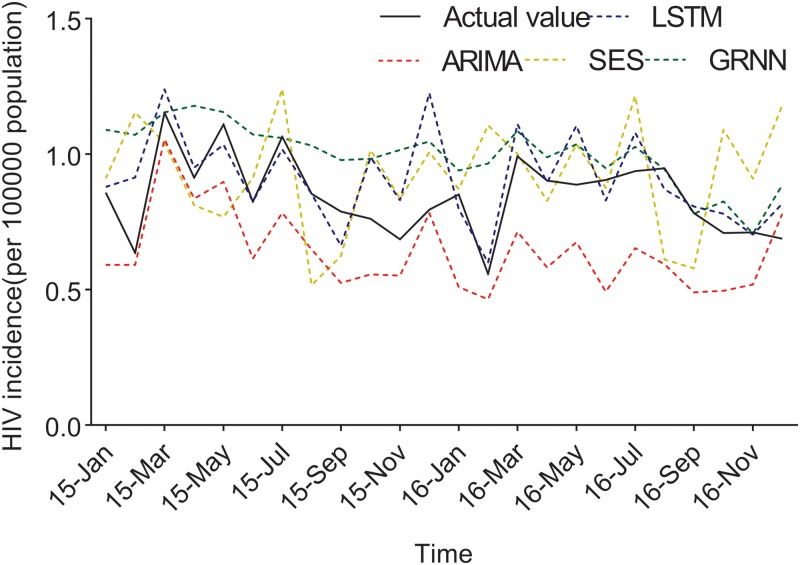
The forecasting curves of the optimal LSTM and other models as well as the actual HIV incidence series. Comparison of LSTM model and other models. LSTM, the long short-term memory neural network model; ARIMA, the autoregressive integrated moving average model. The black line means the actual data, the blue dashed line means the predictive data via the LSTM model, the red dashed line means the predictive value via the ARIMA model, the yellow dashed line means the predictive value via the SES model, while the green dashed line means the predictive value via the GRNN model. Compared with ARIMA, SES and GRNN, the predicted value of LSTM was closer to the actual value.

A multiple neural network architecture which relied on the LSTM layer was used to predict the HIV incidence in 2015 and 2016 using Python programming. HIV incidence data from 2005 to 2014 were used as the training set to construct the LSTM models, and the 2015 data were applied as test sets to evaluate the fitting capacity of the LSTM models. In the same way, the incidence in 2016 was predicted by the data from 2005 to 2015. Before setting up the LSTM forecasting system, the parameters (*N*) were set as shown in Figure 3. The MSE in different *N* values were then determined using HIV incidence in 2015 and 2016, respectively (Fig. 3). The LSTM model when the value of *N* was 12 had the lowest MSE value, and it was selected as the optimal model (Fig. 3). The forecasting curve of the optimal LSTM model and the actual HIV incidence curve are shown in Figure 4.

The predicted values of the optimal LSTM and ARIMA models were compared with the actual HIV incidence in Guangxi in 2015 and 2016, respectively. As shown in Figure 4, forecasting results by the LSTM model were closer to the actual values than those of ARIMA, GRNN and ES models. Furthermore, four performance metrics (MSE, root mean square error (RMSE), mean absolute error (MAE) and mean absolute percentage error (MAPE)) of the LSTM model were all lower than those of the other models (Table 1). Therefore, the LSTM neural network model was superior in predicting the incidence of HIV in Guangxi.

**Table 1.** Performance of LSTM and the other models in 2015 and 2016

	2015				2016			
	MSE	RMSE	MAE	MAPE	MSE	RMSE	MAE	MAPE
LSTM	0.0308	0.1755	0.1231	0.1588	0.0026	0.4189	0.0103	0.0966
ARIMA	0.0357	0.1888	0.1672	0.1925	0.0030	0.4345	0.0139	0.4926
GRNN	0.2005	0.2612	0.0557	0.2359	0.2100	0.1983	0.0489	0.2212
SES	0.0640	0.2458	0.2093	0.2577	0.1300	0.3606	0.3201	0.3162
ES	0.0371	0.1927	0.1663	0.1941	0.1118	0.3344	0.2972	0.2859

MSE, mean square error; RMSE, root mean square error; MAE, mean absolute error; MAPE, mean absolute percentage error.

### Construction of GRNN models

The time series from January 2005 to December 2015 was selected to develop the network. Incidences of November 2016 and December 2016 were selected as the testing samples and the rest were used to train the network. The optimal *N* value of GRNN was 18; namely, a basic model with 18-dimensional input and one-dimensional output showed the minimum RMSE. The forecasting curve of the optimal GRNN model and the actual HIV incidence curve are shown in Figure 4 and the four performance metrics (MSE, RMSE, MAE and MAPE) are shown in Table 1.

### Construction of ES models

ES and seasonal ES methods were developed by JMP 13. As shown in Supplementary Table S2, the *R*^2^ of the seasonal ES model was higher, and the AIC and SBC were lower. The estimation of the parameters of these methods is shown in Supplementary Table S5. MSE, RMSE, MAE and MAPE of the seasonal ES model were all higher than other models.

## Discussion

The HIV epidemic is still severe in Guangxi, and it is necessary to establish an effective monitoring network for the prevention and control of HIV. In the present study, various models for analysing the time series: LSTM, ARIMA and ES were established using the 10-year historical HIV incidence data, and their predictive abilities were compared. We found that the LSTM model had better predictive ability than did ARIMA and other models, which is largely consistent with the results reported by others in predicting the influenza outbreaks [32].

Different models have their own merits and faults. The ARIMA model includes a moving average process, an autoregressive moving average process, an autoregressive moving average process and an ARIMA process according to the different parts of the regression and whether the original data are stable. The model is very simple and requires only endogenous, rather than exogenous, variables. But the limitations of ARIMA model were shown to be as follows: (1) the time series must be stationary or stable after difference; and (2) only linear relationships could be captured [11, 29]. In our study, the ARIMA model showed a large difference in predicting HIV incidences in 2015 and 2016 (Fig. 4). Although ARIMA is a classic model that is widely used to predict the initiation and epidemic progression of infectious diseases, the reason might be that the HIV incidence in Guangxi exhibits a seasonal tendency. Besides, the incidence increased slowly from 2005 to 2011, and the epidemic situation from 2011 to 2015 showed a slow and seasonal decline. However, the LSTM model can model the data of a time series, particularly the long-term data, and automatically determine the optimal time lag [33]. Like most RNNs, the LSTM network is universal, because if enough network units are provided, it can compute anything that the conventional computer can, as long as the proper weight matrix is provided. An LSTM network is well-suited to process and predict the time series when there are very long time lags of unknown size. Thus, LSTM is a more effective and extensible learning model for continuous data than is ARIMA. The GRNN model was developed as a new potential tool for infectious disease incidence prediction. It is characterised by a fast convergence and good non-linear approximation performance based on a radial basis network. However, because each test sample needs to be calculated, the GRNN model has to store all the training samples, which increases the spatial complexity [26]. In our study, the GRNN model had lower performance metrics than the other models, which means it is not an outstanding model to predict the incidence of HIV in Guangxi. ES is also a widely used method for analysing the time series. It does not discard the data in the past, and only gradually decreases the degree of influence [30]. Briefly, the weight gradually converges to zero. Nevertheless, ES lacks the ability to identify the transition of data, and the long-term prediction is poor. In this study, the seasonal ES method showed a large difference in predicting the incidence because the tendency of the original data was intricate.

The characteristics of the HIV/AIDS epidemic in Guangxi are shown to be as follows: (1) the number of cases, diseases and the ratio of late detection were overall high; (2) the proportion of farmers and student cases was increasing year by year, the proportion of individuals over 50 years of age was obviously rising, and the proportion of heterosexual transmission was over 92% in the last 4 years; and (3) a large number of local cases, and new findings and reports indicated that the morbidity had already shifted from high-risk groups to the general population [34]. From 2006 to 2010, the HIV/AIDS entered the stage of the widespread epidemic, in which HIV-1 transmission in Guangxi was dominated by heterosexual transmission, and the tendency from high-risk groups to the general population was obvious. Then, Guangxi accessed the implementation stage of the HIV-1 prevention project from 2011 to 2016, and the fluctuation of HIV incidence declined slowly. This is consistent with the trend in China.

Although the effectiveness of the LSTM model in this study is sufficient and the model is feasible, there are still small deviations between the predicted results and the actual values. The source of these differences might come from a number of influencing factors, including the environmental and behavioural factors, and the intensity of intervention in different areas, etc. [35, 36]. After all, for the LSTM model, time series is the only variable, and other influencing factors could not be included in the model. In addition to the influencing factors mentioned above, there is also a special factor for the HIV/AIDS epidemic in Guangxi, the foreign immigrants and tourists. Guangxi is a border province in China, and is geographically close to the Association of Southeast Asian Nations (ASEAN) countries. The number of immigrants and tourists has increased greatly in recent years, especially those from neighbouring Vietnam [37, 38]. Some studies have shown that female sex workers from Vietnam have spread Vietnam's endemic HIV-1 strains to Guangxi and contributed to the local epidemic [39, 40]. However, this contribution is actually in a node and could not be reflected in the LSTM model. Overall, although the LSTM model in this study has a relatively good effect and predictive ability, more advanced disease forecast models that can include multiple factors need further development in the near future, to make the forecasting more precise and closer to the truth.

## Conclusions

In this study, four types of models, LSTM, ARIMA, GRNN and ES, were established and their predictive abilities compared. The LSTM model was a better predictive model than the others in forecasting the HIV incidence. Establishing an LSTM model is important for the monitoring and control of the local HIV epidemic in Guangxi, China.

## Supplementary material

For supplementary material accompanying this paper visit https://doi.org/10.1017/S095026881900075X. click here to view supplementary material

## References

[1] Xing J (2014) HIV/AIDS epidemic among older adults in China during 2005–2012: results from trend and spatial analysis. Clinical Infectious Diseases 59, 53–60. 10.1093/cid/ciu214 PMC4990827 24700658

[2] China CDC (2018) Update on the AIDS/STD epidemic in China in January, 2018. Chinese Journal of AIDS & STD 24, 219.

[3] Zhang C (2014) Prevalence of HIV, syphilis, and HCV infection and associated risk factors among male clients of low-paying female sex workers in a rural county of Guangxi, China: a cross-sectional study. Sexually Transmitted Infections 90, 230–236. 24482489 10.1136/sextrans-2013-051275 PMC6712973

[4] Willis SJ (2018) Chronic hepatitis C virus infection and subsequent HIV viral load among women with HIV initiating antiretroviral therapy. AIDS (London, England) 32, 653–661. 10.1097/QAD.0000000000001745 PMC6024258 29334550

[5] WHOCSR (2004) WHO Recommended Surveillance Standards, 2nd Edn. WHO Available at http://www.who.int/csr/resources/publications/surveillance/whocdscsrisr992.pdf (Accessed 17 June 2012).

[6] Lyu P (2016) Discussion of HIV control and prevention strategies. Chinese Journal of Preventive Medicine 50, 841–845. 27686758 10.3760/cma.j.issn.0253-9624.2016.10.001

[7] Xu Q (2017) Forecasting influenza in Hong Kong with Google search queries and statistical model fusion. PLoS ONE 12, e0176690. 28464015 10.1371/journal.pone.0176690 PMC5413039

[8] Goodman KE (2016) A clinical decision tree to predict whether a bacteremic patient is infected with an extended-spectrum beta-lactamase-producing organism. Clinical Infectious Diseases 63, 896–903. 27358356 10.1093/cid/ciw425 PMC5019284

[9] Zeng H (2016) Convolutional neural network architectures for predicting DNA-protein binding. Bioinformatics (Oxford, England) 32, 121–127. 10.1093/bioinformatics/btw255 PMC4908339 27307608

[10] Box GE and Jenkins GM (1976) Time series analysis: forecasting and control. Journal of Time 31, 238–242.

[11] Zheng YL (2015) Forecast model analysis for the morbidity of tuberculosis in Xinjiang, China. PLoS ONE 10, e0116832. 25760345 10.1371/journal.pone.0116832 PMC4356615

[12] Zeng Q (2016) Time series analysis of temporal trends in the pertussis incidence in Mainland China from 2005 to 2016. Scientific Reports 6, 32367. 27577101 10.1038/srep32367 PMC5006025

[13] Wu C (2019) Modeling and estimating aboveground biomass of *Dacrydium pierrei* in China using machine learning with climate change. Journal of Environmental Management 234, 167–179. 30620924 10.1016/j.jenvman.2018.12.090

[14] Kim Y (2018) A novel approach to predicting human ingress motion using an artificial neural network. Journal of Biomechanics 84, 27–35. 30558910 10.1016/j.jbiomech.2018.12.009

[15] Guan P (2018) Trends of reported human brucellosis cases in mainland China from 2007 to 2017: an exponential smoothing time series analysis. Environmental Health and Preventive Medicine 23, 23. 29921215 10.1186/s12199-018-0712-5 PMC6010161

[16] Lore KG (2018) A deep learning framework for causal shape transformation. Neural Network 98, 305–317. 10.1016/j.neunet.2017.12.003 29301111

[17] Hochreiter S and Schmidhuber J (1997) Long short-term memory. Neural Computation 9, 1735–1780. 9377276 10.1162/neco.1997.9.8.1735

[18] Chen L (2017) Application of LSTM networks in short-term power load forecasting under the deep learning framework. Electric Power Information & Communication Technology 15, 8–11.

[19] Donahue J (2017) Long-term recurrent convolutional networks for visual recognition and description. IEEE Transactions on Pattern Analysis & Machine Intelligence 39, 677–691. 27608449 10.1109/TPAMI.2016.2599174

[20] Vinyals O (2014) Show and tell: a neural image caption generator. IEEE Conference on Computer Vision & Pattern Recognition (version 1) 3156–3164.

[21] Lin Y (2015) Application of an autoregressive integrated moving average model for predicting injury mortality in Xiamen, China. BMJ Open 5, e008491. 10.1136/bmjopen-2015-008491 PMC4679986 26656013

[22] Dvornek NC (2017) Identifying autism from resting-state fMRI using long short-term memory networks. International Workshop on Machine Learning in Medical Imaging 10541, 362–370. 10.1007/978-3-319-67389-9_42 PMC5669262 29104967

[23] Greff K (2017) LSTM: a search space odyssey. IEEE Transactions on Neural Networks & Learning Systems 28, 2222–2232. 27411231 10.1109/TNNLS.2016.2582924

[24] Gers FA (2000) Learning to forget: continual prediction with LSTM. Neural Computation 12, 2451–2471. 11032042 10.1162/089976600300015015

[25] Specht DF (1991) A general regression neural network. IEEE Transactions on Neural Networks & Learning Systems 2, 568–576. 10.1109/72.97934 18282872

[26] Ghritlahre HK and Prasad RK (2018) Exergetic performance prediction of solar air heater using MLP, GRNN and RBF models of artificial neural network technique. Journal of Environmental Management 223, 566–575. 29975883 10.1016/j.jenvman.2018.06.033

[27] Wang H (2018) Time-series analysis of tuberculosis from 2005 to 2017 in China. Epidemiology & Infection 146, 935–939. 29708082 10.1017/S0950268818001115 PMC9184947

[28] Singh KP (2013) Predicting acute aquatic toxicity of structurally diverse chemicals in fish using artificial intelligence approaches. Ecotoxicology & Environmental Safety 95, 221–233. 23764236 10.1016/j.ecoenv.2013.05.017

[29] Wei W (2016) Application of a combined model with autoregressive integrated moving average (ARIMA) and generalized regression neural network (GRNN) in forecasting hepatitis incidence in Heng County, China. PLoS ONE 11, e0156768. 27258555 10.1371/journal.pone.0156768 PMC4892637

[30] Ke G (2016) Epidemiological analysis of hemorrhagic fever with renal syndrome in China with the seasonal-trend decomposition method and the exponential smoothing model. Scientific Reports 6, 39350. 27976704 10.1038/srep39350 PMC5157041

[31] Pereira A (2004) Performance of time-series methods in forecasting the demand for red blood cell transfusion. Transfusion 44, 739–746. 15104656 10.1111/j.1537-2995.2004.03363.x

[32] Zhang J and Nawata K (2017) A comparative study on predicting influenza outbreaks. Bioscience Trends 11, 533–541. 29070762 10.5582/bst.2017.01257

[33] Ordonez FJ and Roggen D (2016) Deep convolutional and LSTM recurrent neural networks for multimodal wearable activity recognition. Sensors (Basel Switzerland) 16, 1–25. 10.3390/s16010115 PMC4732148 26797612

[34] Ge X (2017) Analysis on epidemiological characteristics and trends of HIV/AIDS in Guangxi during 2010–2015. Chinese Journal of AIDS & STD 24, 864–866.

[35] Harrison A (2015) Sustained high HIV incidence in young women in Southern Africa: social, behavioral, and structural factors and emerging intervention approaches. Current HIV/AIDS Reports 12, 207–215. 25855338 10.1007/s11904-015-0261-0 PMC4430426

[36] Alvarez-Uria G (2012) Trends and risk factors for HIV infection among young pregnant women in rural India. International Journal of Infectious Diseases 16, 121–123. 10.1016/j.ijid.2011.10.003 22153000

[37] Bo H and Yufang S (2014) HIV/AIDS-related high risk behaviors among Vietnamese cross-border floating population in the frontiers of Guangxi province. Journal of Applied Preventive Medicine 20, 6–10.

[38] Huo JL (2016) High risk behaviors of foreign HIV/AIDS patients in China-Vietnam border. Modern Preventive Medicine 43, 4378–4380.

[39] Zhu J (2012) The potential risk factors analysis of HIV/STD infection in Vietnamese cross-border female sex workers. Journal of Kunming Medical University 10, 145–148.

[40] Wang J (2015) Analysis of HIV correlated factors in Chinese and Vietnamese female sex workers in Hekou, Yunnan Province, a Chinese Border Region. PLoS ONE 10, e0129430. 26053040 10.1371/journal.pone.0129430 PMC4459989

