# Supplementary material for: Application of a long short-term memory neural network: a burgeoning method of deep learning in forecasting HIV incidence in Guangxi, China
Source: Epidemiol Infect. 2019 May 9;147:e194. doi: 10.1017/S095026881900075X (PMC6518582; doi:10.1017/S095026881900075X)
Supplement: Supplementary file 1 [file S095026881900075Xsup001.zip › S095026881900075Xsup001/Supplementary_Tables.docx]

| Supplementary Table S1. The ADF test of the transformed HIV incidence series | | | | |
| --- | --- | --- | --- | --- |
|  |  | | **t-statistic** | **P-value** |
| Augmented Dickey-Fuller test statistic | | | -19.032 | <0.0001 |
| Test critical values | | 1% level statistic | -2.58287 |  |
|  | | 5% level statistic | -1.9433 |  |
|  | | 10% level statistic | -1.61509 |  |

| **Supplementary Table S2. The AIC, SBC, and R2 of the three appropriate ARIMA and ES models** | | | |
| --- | --- | --- | --- |
| **Model** | **AIC** | **SBC** | **R^2^** |
| ARIMA(2,1,0)(1,1,2)_12_ | -0.9811 | -0.8547 | 0.6341 |
| ARIMA(2,1,1)(0,1,2)12 | -0.8534 | -0.759 | 0.5906 |
| ARIMA(0,1,0)(2,1,2)12 | -0.7749 | -0.6673 | 0.5709 |
| SES | -23.5757 | -17.8253 | 0.4579 |
| ES | -8.7679 | -5.8051 | 0.4161 |
| ARIMA: the autoregressive integrated moving average; SES: seasonal exponential smooth; ES: exponential smooth; AIC: Akaike information criterion; SBC: Schwarz Bayesian information criterion. | | | |

| **Supplementary Table S3. Estimating parameters of the ARIMA (1, 1, 2) (0, 1, 2)12 model in 2015** | | | | |
| --- | --- | --- | --- | --- |
| **Variable** | **Coefficlent** | **Stu-Error** | **t-statistic** | **P-value** |
| AR(1) | -0.9676 | 0.0269 | -35.9657 | <0.0001 |
| MA(1) | 0.3849 | 0.0798 | -4.8253 | <0.0001 |
| MA(2) | -0.5959 | 0.0776 | -7.6779 | <0.0001 |
| SMA(12) | -0.8889 | 0.0198 | -44.9611 | <0.0001 |
| SMA(24) | 0.8328 | 0.0294 | 28.3165 | <0.0001 |
| Estimating parameters of the ARIMA (1, 1, 2) (0, 1, 2)12 model in 2015. ARIMA: the autoregressive integrated moving average; MA(1): Moving average, lag1; MA(2): Moving average, lag2; SAR(12): Seasonal moving average, lag12; SMA(12) : Season Moving average, lag12; SMA(24): Season Moving average, lag24. | | | | |

| **Supplementary Table S4. Estimating parameters of the ARIMA (2, 1, 0) (1, 1, 2)12 model in 2016** | | | | |
| --- | --- | --- | --- | --- |
| **Variable** | **Coefficlent** | **Stu-Error** | **t-statistic** | **P-value** |
| AR(1) | -0.6356 | 0.0932 | -6.8164 | <0.0001 |
| AR(2) | -0.3696 | 0.0937 | -3.946 | 0.0001 |
| SAR(12) | 0.6146 | 0.0638 | 9.6391 | <0.0001 |
| SMA(12) | -1.568 | 0.0239 | -65.6161 | <0.0001 |
| SMA(24) | 0.838 | 0.0189 | 44.2785 | <0.0001 |

| **Supplementary Table S5. Parameters estimation of SES and ES** | | | | |
| --- | --- | --- | --- | --- |
|  | **Estimator** | **SE** | **t-statistic** | **P-value** |
| **SES** |  |  |  |  |
| Horizontal smoothing weight | 0.2689 | 0.0542 | 4.96 | <0.0001 |
| Seasonal smoothing weight | 0.8164 | 0.1719 | 4.75 | <0.0001 |
| **ES** |  |  |  |  |
| Horizontal smoothing weight | 0.4789 | 0.0828 | 5.78 | <0.0001 |
| SES: seasonal exponential smooth model; ES: exponential smooth model. | | | | |
