# Supplementary figures and images for: Application of a long short-term memory neural network: a burgeoning method of deep learning in forecasting HIV incidence in Guangxi, China
Source: Epidemiol Infect. 2019 May 9;147:e194. doi: 10.1017/S095026881900075X (PMC6518582; doi:10.1017/S095026881900075X)

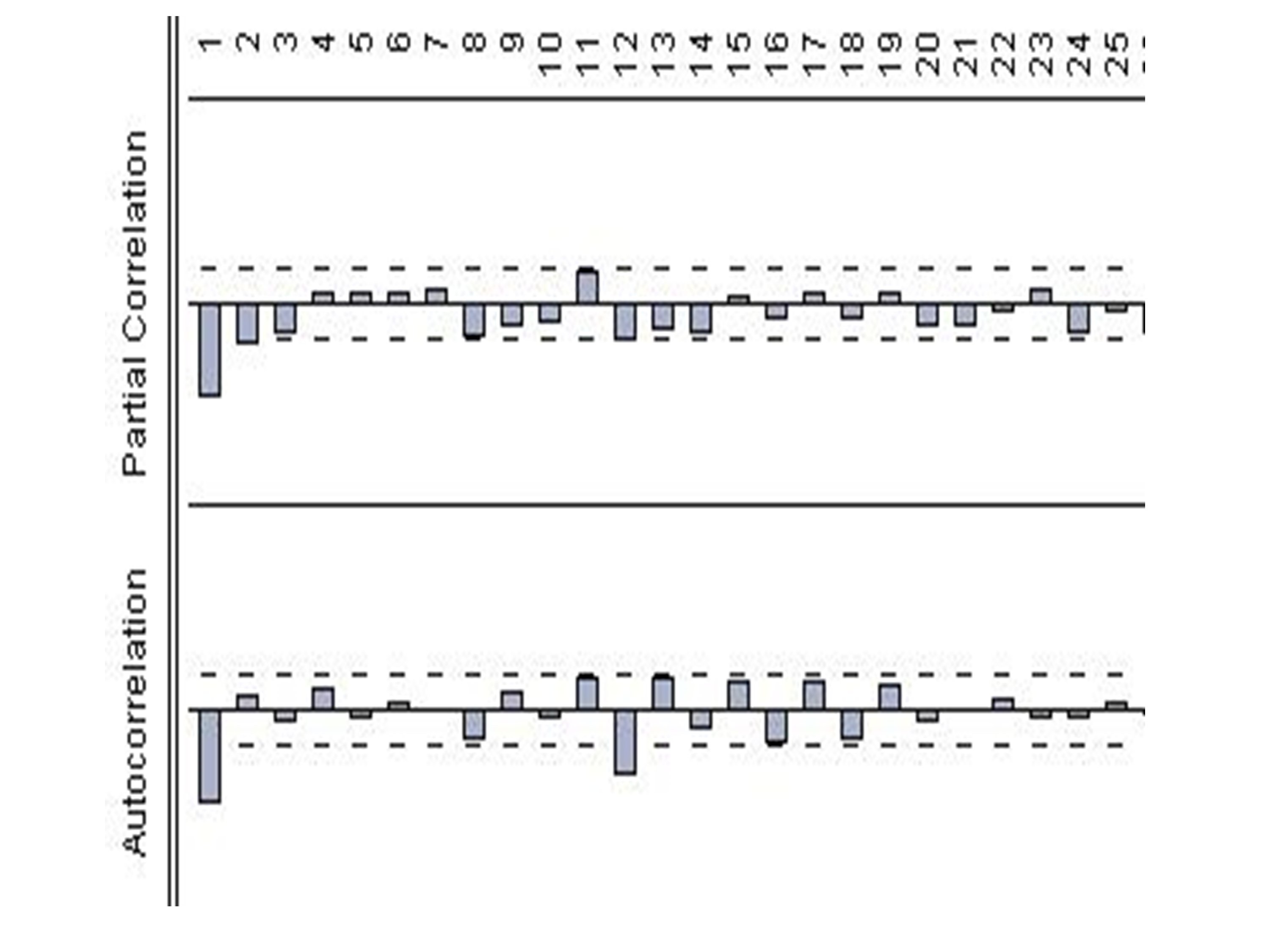

Supplement: Supplementary file 1 [file S095026881900075Xsup001.zip › S095026881900075Xsup001/Supplementary_Figure_S1._The_ACF_and_PACF_graphs__of_transformed_HIV_incidence_series.tif]
